# Regular Physical Exercise Adherence Scale (REPEAS): a new instrument to measure environmental and personal barriers to adherence to regular physical exercise

**DOI:** 10.1186/s12889-023-17438-1

**Published:** 2023-12-13

**Authors:** Fábio Henrique Ferreira Pereira, Aldair Darlan Santos-de-Araújo, André Pontes-Silva, Renan Shida Marinho, Adriana Sanches Garcia-Araújo, Audrey Borghi-Silva, Maria Cláudia Gonçalves, Rita de Cássia Mendonça de Miranda, Jhonata Botelho Protazio, Cezar Augusto Brito Pinheiro, Almir Vieira Dibai-Filho, Daniela Bassi-Dibai

**Affiliations:** 1 Postgraduate Program in Environment, Universidade Ceuma, São Luís, Maranhão Brazil; 2 Department of Physical Therapy, Postgraduate Program in Physical Therapy, Universidade Federal de São Carlos, São Carlos, São Paulo Brazil; 3 Postgraduate Program in Physical Education, Universidade Federal do Maranhão, São Luís, Maranhão Brazil; 4 Postgraduate Program in Adult Health, Universidade Federal do Maranhão, São Luís, Maranhão Brazil; 5 Postgraduate Program in Management of Health Services and Programs, Universidade Ceuma, São Luís, Maranhão Brazil; 6 Departament of Physical Therapy, Universidade Ceuma, São Luís, Maranhão Brazil; 7 Postgraduate Program in Dentistry, Universidade Ceuma, São Luís, Maranhão Brazil; 8 Programa de Pós-Graduação em Meio Ambiente, Universidade Ceuma, Rua Josué Montello, 1, Jardim Renascença. CEP 65075-120, São Luís, Maranhão Brazil

**Keywords:** Sedentary behavior, Physical exercise, Motivation

## Abstract

**Objective:**

To create, develop, and validate a scale that identifies the environmental and personal barriers that make it difficult to adhere to the practice of physical exercise on a regular basis in a population of Brazilian adults.

**Methods:**

We include adult individuals, aged 18–59 years, practitioners or former practitioners of physical exercise, with Brazilian Portuguese as their mother tongue. In the development and validation phases of the process, 6 specialists in the field of the health assessed the content validity: firstly, the specialists were asked to freely list the questions they would ask to investigate the barriers to adherence to regulating physical activity. Secondly, after compiling all the suggestions listed and eliminating suggestions with similar content, the items suggested in the first round were sent to the specialists so that an evaluation of all questions using a 5-point Likert scale and the content validity coefficient was calculated. We then evaluated the structural validity, construct validity, reliability, internal consistency, and ceiling and floor effects of the Regular Physical Exercise Adherence Scale (REPEAS).

**Results:**

Sixteen items were proposed to measure the factors that make it difficult to adhere to the regular practice of physical exercise. The internal structure of the REPEAS initially tested was based on the theoretical proposal of creating the instrument with two domains. After the structural analysis, we used the modification indices to identify the redundant items of the instrument. Consequently, the final version of the REPEAS after factor analysis had 12 items. Thus, the structure with 2 domains and 12 items presented adequate fit indices. With regard to construct validity, the REPEAS scores were compared in two distinct groups: irregular practitioners/ex-practitioners versus regular practitioners of physical exercise, in which a significant difference could be observed between groups (*p* < 0.001) for both the domains. Acceptable reliability was observed for the environment and personal domains, with ICC values of 0.86 and 0.94, in the same order. For internal consistency, Cronbach's alpha value was 0.908 (environmental domain) and 0.915 (personal domain), these values being adequate for the REPEAS.

**Conclusion:**

The REPEAS is a scale with a valid two-dimensional internal structure, consisting of 12 items, reliable and with a valid construct, which supports its use in the clinical, epidemiological, and research contexts in Brazil.

**Supplementary Information:**

The online version contains supplementary material available at 10.1186/s12889-023-17438-1.

## Introduction

Physical inactivity is considered one of the largest modifiable risk behaviors, being the fourth largest risk factor contributing to death [1]. There is strong evidence that over the past 40 years, the level of physical activity (PA) of the population has declined sharply in countries with higher per capita income, with middle- and low-income countries expected to follow this trend [2, 3]. PA is considered to be any behavior that involves body movements in everyday life that result in muscle stimulation and energy expenditure and that is performed intentionally (e.g., walking, running, or climbing stairs) [4]. Physical exercise (PE) is programmed and well-structured practice aimed at improving physical components such as muscle structure, flexibility, and balance [5].

Regular PE is part of a healthy lifestyle, with several cross-sectional studies consolidating the overall reduced risk of cardiovascular disease and cardiac events associated with habitual or leisure-time PE [6, 7]. The regular practice of PE can be considered a non-pharmacological therapeutic modality to reduce cardiovascular morbidity and mortality, thanks to the cardiovascular benefit induced by exercise [8, 9]. Studies have shown the positive effects of PE on mental health problems [10, 11], in addition to improving the cognitive function in the population and reducing the risk of developing cognitive impairment [12–14]. PE can produce several physiological and mechanical changes in the body, which in turn can reduce stress levels, resulting in protection against negative health effects [15], in addition to improving mood and positive affect [16].

Considering the environmental factors that are related to the practice of PE, a higher density of green spaces such as parks and squares is associated with higher levels of PA [17]. The provision of places such as forests, parks and squares, which are generally free of charge, expands access to space and structures for the practice of PA. In addition, a nationwide study found that 53.4% of participants in the Midwest region of Brazil reported engaging in leisure-time PA at least once a week in a public space near their home [18]. In turn, moderate-intensity PA is practiced at any time of day, but vigorous PA is more common at night [19].

When attempting to influence an individual to adopt a physically active lifestyle, it is necessary to consider the individual's particular circumstances, whether financial and/or social. In addition, people are physically active as a result of the support/influence of family, friends, and/or colleagues for the practice of PA [20]. Considering the importance of the regular practice of PE for the general population, the creation of an instrument that examines the barriers to adherence to PE is justified as a way to subsidize measures to confront sedentary behavior. As such, the present study aimed to create, develop, and validate a scale that identifies the environmental and personal barriers that make it difficult to adhere to the practice of physical exercise on a regular basis in a population of Brazilian adults, namely, the Regular Physical Exercise Adherence Scale (REPEAS).

## Material and methods

### Study design and ethical aspects

This is a cross-sectional study to validate a questionnaire carried out in accordance with the guidelines of the COnsensus-based Standards for the selection of health Measurement INnstruments (COSMIN) [21]. The research was carried out in two states of the Brazilian territory, Maranhão and São Paulo, through the application of an online form and approved by the Research Ethics Committee of the Universidade Ceuma (protocol number 5.328.899) and conducted according to Declaration of Helsinki. All patients were informed about the purpose and procedures of the study through a previous description of the purpose of the research on the first page of the online form and at the end the volunteers were invited to sign the free and informed consent form to then be directed to the consequent pages that contained the questions necessary for the development of the study.

### Sample size

The sample consisted of adult individuals, of both genders, aged between 18 and 59 years, regular or irregular practitioners or former practitioners of physical exercise, with Brazilian Portuguese as their mother tongue. Volunteers were recruited via social media (Instagram and Facebook, Meta, Menlo Park, CA, USA) and mobile messaging apps (WhatsApp, Meta, Menlo Park, CA, USA). Following the COSMIN guidelines, the sample size was calculated taking into account the number of items in the instrument multiplied by 7. Considering the 16 items initially proposed for the REPEAS, the almost general minimum size to be achieved was 112 participants [21].

Those individuals who presented any impossibility to answer the proposed questionnaires, medical diagnosis of severe cognitive or psychiatric alteration, and medical contraindication for performing physical exercises were excluded.

### Data collect

Data collection took place via an online form (Google Forms, Mountain View, CA, USA). Initially, a collection of personal, professional, anthropometric and healthy habits data was carried out. Then it was applied to REPEAS. In addition, to characterize the level of habitual physical activity, the Baecke Habitual Physical Activity Questionnaire (BHPAQ) [22] was applied.

### Content validity

To create the scale, content validity was performed using the Delphi method [23, 24]. To reach the initial version of the scale, two rounds were carried out with 6 specialists in the field of health (physiotherapist, biologist, and bachelor of physical education): in the first round, the specialists were asked to freely list which questions they would ask to investigate the barriers that hinder adherence to the regular practice of physical exercise. In the second round, after compiling all the suggestions listed and excluding suggestions with similar content, the items suggested in the first round were sent to the specialists so that an evaluation could be issued for two questions using a 5-point Likert scale (1 – not at all; 2 – a little; 3 – reasonably; 4 – a lot; and 5 – totally): question 1 – “How important is this item to be considered in the assessment of the barriers that make it difficult to adhere to the regular practice of physical exercise?”; Question 2 – “How clear and easy is this item to understand?”. After that, the content validity coefficient was calculated [25, 26].

### Structural validity

To verify the internal structure of the REPEAS, confirmatory factor analysis (CFA) was used [27]. The theoretical assumption is that the REPEAS has a structure with two domains: environmental factors and personal factors.

### Construct validity

In order to validate the construct, the score of each REPEAS domain was compared between two recognizably different groups: regular practitioners of physical exercise versus ex-practitioners/irregular practitioners of physical exercise.

### Reliability and internal consistency

For the analysis of test–retest reliability and internal consistency, a subsample of 75 participants was used who answered the REPEAS in two moments, with an interval of 7 days between assessments [28].

### Regular Physical Exercise Adherence Scale (REPEAS)

The REPEAS is the target tool of this study. This scale aims to assess the factors that hinder adherence to the practice of regular physical exercise. The scale consists of a list of environmental and personal factors that may hinder adherence to regular physical exercise. The respondent must indicate on a scale from 0 to 10 the answer option that best indicates these situations, in which 0 means “Does not make it difficult to practice physical exercise” and 10 means “It makes it very difficult to practice physical exercise”. For the total score, the values of the answers given to the items must be added and divided by the number of items answered, generating a score that varies from 0 to 10. After that, the value must be multiplied by 10, generating a score of 0 to 100. The higher the score, the greater the barriers to adherence to physical exercise.

### Baecke habitual physical activity questionnaire

The Baecke Habitual Physical Activity Questionnaire (BHPAQ) is a self-administered instrument based on self-report that assesses physical activity in the last 12 months. It consists of 16 items, divided into three domains: physical activity during occupation (items 1 to 8), physical activity in sport during leisure time (items 9 to 12) and physical activity during leisure time without sport (items 13 to 16). To calculate the final score, the separate domain must be considered. The total score ranges from 1 to 5, the higher the score, the greater the usual physical activity. The BHPAQ has been adapted and validated for Brazilian Portuguese [22].

### Statistical analysis

For content validity, the calculation of the content validity coefficient was used [25, 26]. Initially, the average of the scores given (scale from 1 to 5) by the specialists to each of the REPEAS items was calculated in terms of clarity and content. After that, the average of the grades given was divided by the maximum possible value that the item could reach (i.e., 5). Then, from the value resulting from the division, the error value was subtracted. To reach the error value, the value 1 was divided by the number of specialists (i.e., 6) and this value was raised to the same number of specialists. Therefore, at the end of these arithmetic procedures, the content validity coefficient value was obtained, with a value equal to or greater than 0.80 being acceptable; items with values below this cutoff point were excluded.

For structural validity, CFA was performed in the R Studio software (Boston, MA, USA), using the lavaan and semPlot packages. The REPEAS is scored on a Likert scale (ordinal data). Thus, the CFA was performed with the implementation of a polychoric matrix and the robust diagonally weighted least squares (RDWLS) extraction method. The fit of the model was evaluated using the root mean square error of approximation (RMSEA) indices with a confidence interval (CI) of 90%, comparative fit index (CFI), Tucker-Lewis index (TLI), standardized root mean square residual (SRMR) and chi-square/degrees of freedom (DF).

In the present study, values greater than 0.90 were considered adequate for CFI and TLI, and values less than 0.08 were considered adequate for RMSEA and SRMR. Values below 3.00 were considered adequate in the interpretation of the chi-square/DF [29, 30]. In the CFA, factor loadings equal to or greater than 0.30 were considered adequate for the domain. For REPEAS refinement, we used modification indices > 10 to identify redundant items and excluded items with lower factor loadings in each paired analysis [31]. In addition, the internal consistency was calculated using Cronbach's alpha to identify whether there are redundant or heterogeneous items in the questionnaire. Cronbach's alpha values considered adequate vary between 0.70 and 0.95 [32].

Reliability was evaluated based on a test–retest model, using the intraclass correlation coefficient (ICC), considering a value equal to or greater than 0.75 as the acceptability cutoff point [33]. Furthermore, we calculated the measurement standard error (SEM) and minimum detectable difference (MDD) [28]. Ceiling and floor effects were evaluated in the present study. By definition, these effects occur when a number of study participants (more than 15%) reach the minimum or maximum values of the total questionnaire score.

In order to determine the validity of the construct, a comparison was made between recognizably different groups (regular practitioners of physical exercise versus ex-practitioners/irregular practitioners of physical exercise) using the t test for independent samples. The hypothesis is that there is a significant difference in the scores of the REPEAS domains in the comparisons between the groups [21].

In addition to the *p*-value, we calculated Cohen’s effect size (d value) to test whether the differences between the evaluated regions were clinically relevant [34]. Cohen’s d was used to assess effect sizes when comparing two means (0.2 = small effect, 0.5 = moderate effect, and 0.8 = large effect) [35, 36]. The processing of descriptive analysis was performed using the SPSS software, version 17.0 (Chicago, IL, USA).

## Results

### Content validity

Initially, emails were sent to 10 specialists in the field of physical exercise, health and the environment. Of these, 6 specialists returned to the request of the first round and proposed 16 items to measure the barriers to adherence to the regular practice of physical exercise. Table 1 presents the academic and professional characteristics of the 6 specialists who participated in the study.

**Table 1:** Characteristics of the specialists participating in the study

Specialist	Characteristics
Specialist 1	Physiotherapist for 14 years with a doctorate in rehabilitation and functional performance. He is a university professor of the physical education course and develops research related to assessment measures centered on self-report for different populations. He is an irregular practitioner of physical exercise
Specialist 2	Biologist for 25 years with a doctorate in Biology. She is a university professor of environmental engineering, physiotherapy, nutrition and biomedicine courses, and develops research related to the environment and biotechnology. She is a regular practitioner of physical exercise
Specialist 3	Physiotherapist for 14 years with a doctorate in health sciences. He is a university professor of physiotherapy and medical courses, and develops research related to the application of exercise and other resources as a way of treating chronic disorders. He is a regular practitioner of physical exercise
Specialist 4	Physiotherapist for 20 years with a doctorate in physiotherapy. She is a university professor of the physiotherapy course and develops research related to exercise physiology and functional performance. She is a former exercise practitioner
Specialist 5	Bachelor of Physical Education for 15 years with a doctorate in Physical Education. He is a university professor of physical education and develops research related to exercise physiology and performance. He is a regular practitioner of physical exercise
Specialist 6	Physiotherapist for 17 years with a doctorate in physiotherapy. She is a university professor of the physiotherapy course and develops research related to physical exercise, chronic diseases and assessment measures centered on self-report. She is a former exercise practitioner

For the second round, experts were asked to assign a score from 1 to 5 on content and clarity for each of the 16 items proposed in the first round. Therefore, as shown in Table 2, the content validity coefficient was applied, without the need to exclude items (values ≥ 0.80). Therefore, the REPEAS version after content validity had 16 items.

**Table 2:** Content validity coefficient of the Regular Physical Exercise Adherence Scale (REPEAS) for each item proposed by the specialists

Item	Content	Clarity
1. Climatic factors of the city where you live (for example, excessive heat or cold, rain, low humidity and/or others)	0.8	0.9
2. Absence of suitable public places for the practice of physical exercise close to your residence (for example, squares, parks, fields, beaches and/or others)	1.0	0.9
3. Feeling of insecurity in places close to home (for example, fear of being the victim of robberies, thefts and/or similar)	0.9	1.0
4. Live far from the appropriate places to practice physical exercise in your city	0.9	1.0
5. Difficulty accessing places to practice physical exercise due to the presence of uneven sidewalks, stairs and/or other obstacles	0.8	1.0
6. Lack of time due to professional, educational, family and/or other commitments	1.0	1.0
7. Lack of financial resources to attend private spaces (for example, gyms or clubs)	1.0	1.0
8. Lack of company to practice physical exercise	0.8	1.0
9. Lack of encouragement from family and/or friends	0.8	1.0
10. Limitations of one’s own body (for example, presence of pain or difficulty moving around)	1.0	1.0
11. Laziness, disinterest, discouragement and/or lack of disposition	1.0	1.0
12. Fear of getting injured or hurt	0.9	0.9
13. Lack of equipment, clothing, shoes and/or accessories for the practice of physical exercise	0.9	1.0
14. Lack of monitoring by a professional to advise you on the practice of physical exercise	0.9	1.0
15. Low self-esteem, shame, and/or other preoccupations with one's physical appearance	0.8	1.0
16. Bad experience with physical exercise in the past	0.8	0.9

After that, the questions were transformed into sentences in the first-person singular and 11 response options were added to the scale items, in which 0 represents “Does not make physical exercise difficult” and 10 represents “It makes physical exercise very difficult”. The REPEAS version with 16 items was then applied to 30 former practitioners or irregular practitioners of physical exercise to investigate the degree of understanding of the scale items. Of these participants, 22 (73.33%) were women, with a mean age of 27.57 years (standard deviation [SD] = 8.61), with walking (46.66%) being the sport or physical activity most reported by these participants. We observed 100% comprehension for all REPEAS items.

### Structural validity

This analysis was performed with 114 participants (irregular/ex-practitioners of physical exercise). The internal structure of the REPEAS initially tested was based on the theoretical proposal of creating the instrument with two domains: the environmental factors domain (items 1 to 5) and the personal factors domain (items 6 to 16). Therefore, we identified four inadequate fit indices in the CFA for this initial structure: chi-square/DF = 2.85, CFI = 0.881, TLI = 0.861, RMSEA (90% CI) = 0.128 (0.111, 0.146), SRMR = 0.101.

After that, we excluded item 6 from the REPEAS because it had a factorial load of less than 0.30 and we used the modification indices to identify the redundant items of the instrument. Thus, as shown in Table 3, we paired the items with a modification index greater than 10 and excluded the items with the lowest factor loading, resulting in a REPEAS structure with 12 items: the environmental factors domain (items 1 to 5) and personal factors domain (items 9 to 15).

**Table 3:** Redundant items according to modification indices (MI)

Redundant items	Item Description	MI	factorial load	Item deleted
**Decision 1**				
Item 8	Lack of company to practice physical exercise	40.954	0.461	Item 8
Item 9	Lack of encouragement from family and/or friends		0.606	
**Decision 2**				
Item 12	Fear of getting injured or hurt	31.745	0.754	Item 16
Item 16	Bad experience with physical exercise in the past		0.742	
**Decision 3**				
Item 3	Feeling of insecurity in places close to home (for example, fear of being the victim of robberies, thefts or similar)	11.931	0.808	Item 7
Item 7	Lack of financial resources to attend private spaces (for example, gyms or clubs)		0.701	

**Fig. 1:**
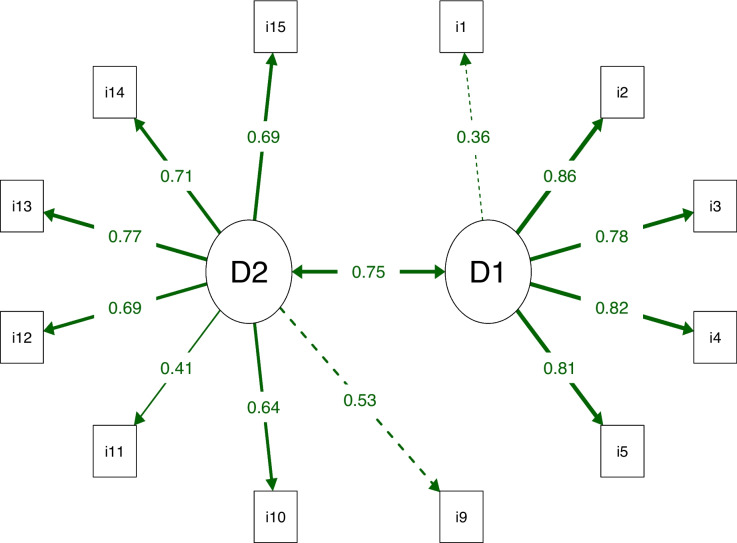
Path diagram of the Regular Physical Exercise Adherence Scale (REPEAS) showing factorial loads greater than 0.30. D1: Environmental factors domain; D2: Personal factors domain. The thicker the line, the greater the factorial load. Dotted lines indicate the first item in the domain

The structure with 2 domains and 12 items presented adequate fit indices: chi-square/DF = 1.63, CFI = 0.973, TLI = 0.966, RMSEA (90% CI) = 0.075 (0.044, 0.102), SRMR = 0.062. Figure 1 shows the factor loadings of the items in their respective domains, with values above the acceptability cutoff point of 0.30. Thus, the final version of the REPEAS with 2 domains and 12 items was established (Additional File 1).

### Sample characterization

We included 228 individuals in the present study, comprising 114 irregular/ex-practitioners of physical exercise and 114 regular practitioners of physical exercise. The characterization of the sample regarding the quantitative variables is shown in Table 4, with a significant difference in value for body mass and BMI in the group of irregular practitioners/ex-practitioners of physical exercise and the sport domain of the BHPAQ in the group of regular practitioners (*p* < 0.05) with a large effect size (d value ≥ 0.8).

**Table 4:** Characterization of the sample of quantitative variables (*n* = 228)

**Variables**	**Group 1 (*n* = 114)**		**Group 2 (*n* = 114)**		***p* value**	
	**Mean**	**Standard deviation**	**Mean**	**Standard deviation**		**d value**
Age (years)	28.02	7.70	28.28	7.96	0.800	0.03
Body mass (kg)	71.25	19.73	66.93	11.42	0.044^a^	0.26
Stature (m)	1.67	0.08	1.67	0.08	0.650	0.00
BMI (kg/m^2^)	25.49	5.73	23.93	3.29	0.013^a^	0.33
Work hours (per day)	7.59	2.88	8.39	4.69	0.119	0.20
Weekly frequency of work (times)	4.86	1.26	5.06	0.92	0.170	0.12
BHPAQ-O (score, 1–5)	2.78	0.61	2.71	0.64	0.483	0.11
BHPAQ-S (score, 1–5)	2.17	0.64	3.06	0.82	< 0.001^a^	1.21^b^
BHPAQ-L (score, 1–5)	2.58	0.65	2.63	0.66	0.513	0.07

Group 1: Irregular practitioners/ex-practitioners of physical exercise; Group 2: Regular practitioners of physical exercise; *BMI* Body mass index, *BHPAQ*-O Occupational domain of the Baecke Habitual Physical Activity Questionnaire, *BHPAQ-S* Sport domain of the Baecke Habitual Physical Activity Questionnaire, *BHPAQ-L* Leisure domain of the Baecke Habitual Physical Activity Questionnaire

^a^Significant difference (independent t-test, *p* < 0.05)

^b^Large effect size (d value ≥ 0.8)

The study included participants residing in two states of the Brazilian territory, Maranhão and São Paulo. We observed that most of the sample was composed of female individuals and strength training was the sport or physical exercise most frequently reported in both groups (Table 5). Comparing the groups, no significant difference was found (*p* > 0.05).

**Table 5:** Characterization of the sample of qualitative variables (*n* = 228)

Variables	Group 1 (*n* = 114)		Group 2 (*n* = 114)		*p* value
	**n**	**%**	**n**	**%**	
Sex					0.075
Male	36	31.57	49	42.98	
Female	78	68.42	65	57.01	
State					0.120
Maranhão	64	56.14	44	38.59	
São Paulo	50	43.85	70	61.40	
Education					0.171
Incomplete primary education	0	0	0	0	
Incomplete primary education	3	2.63	1	0.87	
Incomplete secondary education	3	2.63	2	1.75	
Incomplete secondary education	9	7.89	11	9.64	
Incomplete higher education	37	32.45	23	20.17	
Complete higher education	25	21.92	21	18.42	
Incomplete postgraduate	13	11.40	22	19.29	
Complete postgraduate	24	21.05	34	29.82	
Sport modality					0.111
Hike	31	27.19	9	7.89	
Soccer	8	7.01	8	7.01	
Strength training	42	36.84	73	64.03	
Pilates	3	2.63	2	1.75	
Others	30	26.31	22	19.29	

Group 1: Irregular practitioners/ex-practitioners of physical exercise; Group 2: Regular practitioners of physical exercise

No significant difference (chi-square test, *p* >0.05)

### Construct validity

With regard to construct validity, the REPEAS scores were compared in two distinct groups: irregular practitioners/ex-practitioners versus regular practitioners of physical exercise. We observed a significant difference between groups (*p* < 0.05), so that the construct is valid (Table 6).

**Table 6:** Comparison of the Regular Physical Exercise Adherence Scale (REPEAS) scores between irregular practitioners/ex-practitioners versus regular practitioners of physical exercise (*n* = 228)

Domains	Group 1 (*n* = 114)		Group 2 (*n* = 114)		*p* value	d value
	**Mean**	**Standard deviation**	**Mean**	**Standard deviation**		
Environmental factors (score, 0–100)	49.63	26.80	35.04	25.88	< 0.001^a^	0.533^b^
Personal factors (score, 0–100)	38.61	21.55	22.68	20.08	< 0.001^a^	0.767^b^

Group 1: Irregular practitioners/ex-practitioners of physical exercise; Group 2: Regular practitioners of physical exercise

^a^Significant difference (independent t-test, *p* < 0.05)

^b^Moderate effect size (d value ≥ 0.5)

### Reliability and internal consistency

In the test–retest model, the REPEAS was applied to a sample of 75 individuals. As shown in Table 7, acceptable reliability was observed for the environment and personal domains, with ICC values of 0.86 and 0.94, respectively. For internal consistency, Cronbach's alpha value was 0.908 (environmental domain) and 0.915 (personal domain), these values being adequate for the REPEAS.

**Table 7:** Test–retest reliability and internal consistency of the Regular Physical Exercise Adherence Scale (REPEAS) (*n* = 75)

Measures	Domains	
	**Environmental factors**	**Personals factors**
Test	47.49 (28.24)	36.13 (23.70)
Retest	48.29 (28.41)	37.56 (24.69)
ICC	0.86	0.94
SEM	10.45	6.17
MDD	28.95	17.10
Cronbach's alpha	0.908	0.915

*ICC* Intraclass correlation coefficient, *SEM* Standard error of measurement, *MDD* Minimum detectable difference

### Ceiling e floor effects

We noted that 6 (5.3%) and 2 (1.8%) participants reached the minimum score in the domain environmental factors and personal factors, respectively. No participants reached the maximum score. Thus, ceiling and floor effects were not observed.

## Discussion

This study aimed to develop a new tool and validate it for the adult Brazilian population, with the aim of identifying the environmental and personal barriers that hinder adherence to regular physical exercise in adults (according to the characteristics of this sample presented in the methods and Table 4 and 5), due to the absence in the literature of an ample tool for due purposes. We observed that the REPEAS presents a two-dimensional structure with 12 items after performing the content validity and structural validity. Furthermore, the REPEAS presents a significant difference between two distinct groups (irregular practitioners/ex-practitioners versus regular practitioners of physical exercise), thus validating the construct. Ceiling and floor effects were not observed.

The literature presents some instruments to measure adherence to physical exercise, however, the REPEAS presents specific characteristics that differentiate it. In this context, the proposal for the elaboration of the Exercise Adherence Rating Scale (EARS) [37] was due to the lack of a valid and reliable tool to assess adherence to prescribed home physical exercise, presenting adequate internal consistency (Cronbach’s alpha = 0.810) and reliability (ICC = 0.970). This instrument consists of two scales totaling 16 items, 6 of which are aimed at directly assessing the behavior of adherence to physical exercise.

Currently, the EARS is available in the literature in British English and, recently, it was adapted to Brazilian Portuguese presenting adequate reliability (ICC ≥ 0.8, and internal consistency (Cronbach’s alpha = 0.88) [38]. Similar to the REPEAS, these previous studies showing good measurement properties, including acceptable internal consistency and high test–retest reliability of the EARS. However, the application of the EARS is specific for individuals with chronic low back pain, while the REPEAS can be used in the general population.

The Questionnaire of Barriers to the Practice of Physical Activities in the Elderly [39] is an instrument developed in Portuguese based on the list of perceived barriers to the practice of PA contemplated in the literature. The Questionnaire of Barriers to the Practice of Physical Activities in the Elderly have items similar to REPEAS, such as “lack of safety in the environment”, “unfavorable climatic factors”, and “having had bad experiences with physical exercise”. However, the Questionnaire of Barriers to the Practice of Physical Activities in the Elderly should be used exclusively in patients with Parkinson’s disease.

Another instrument in Portuguese is the Motivation Inventory for Regular Practice of Physical Activity and/or Sport with aim to assess the motivation to regularly practice physical activity and/or sport through more than 100 items. However, its structural validity is inadequate according to most fit indices presented in the study (chi-square/DF = 4.337, goodness-of-fit index = 0.840, adjusted goodness-of-fit = 0.831, RMSEA = 0.068) [40]. In comparison, the Motivation Inventory for Regular Practice of Physical Activity and/or Sport evaluates only the motivation for the regular practice of physical activity and/or sport, while the REPEAS seeks to measure the environmental and personal barriers to adherence to the practice of regular physical exercise, which is the main difference between the aforementioned tools.

In this context, the REPEAS presented some positive characteristics when compared to the instruments mentioned, such as: it presents a smaller number of items, which results in a shorter filling time; it has items that allow an assessment of the general population, that is, it is not limited to a specific population, such as the EARS (for low back pain) and Questionnaire of Barriers to the Practice of Physical Activities in the Elderly (for Parkinson’s disease); it presents two clear and well-defined domains, with a simple and easy-to-interpret score.

This study presents as a limitation the exclusivity of the REPEAS to assess the environmental and personal barriers to adherence to the practice of physical exercise on a regular basis aimed only at adults. Additionally, in the content validity, some health professionals are not experts in physical exercise but experts in the environment, health assessment, or other aspects related to physical exercise. Therefore, future studies should validate this instrument for other populations, such as the elderly and patients with chronic diseases. The REPEAS was developed in Brazilian Portuguese and requires transcultural adaptation for use in other languages. Given the territorial dimension of Brazil and the climatic, socio-cultural, demographic, and economic differences in the country, we also suggest that researchers verify that this validation applies to all regions of Brazil. Finally, we did not evaluate the measurement properties of the REPEAS considering the respondents’ state (Maranhão or São Paulo) due to the small sample size.

## Conclusion

The REPEAS is a scale with a valid two-dimensional internal structure, consisting of 12 items, reliable and with a valid construct, which supports its use in the clinical, epidemiological, and research contexts in Brazil.

### Supplementary Information

**Additional file 1.**

## Data Availability

The data and materials in this article will be made available by the corresponding author upon reasonable justification.

## Abbreviations

PA: Physical Activity
PE: Physical Exercise
REPEAS: Regular Physical Exercise Adherence Scale
COSMIN: Consensus-based Standards for the selection of health Measurement Instruments
BHPAQ: Baecke Habitual Physical Activity Questionnaire
CFA: Confirmatory Factor Analysis
RDWLS: Robust diagonally weighted least squares
RMSEA: Root mean square error of approximation
CFI: Comparative fit index
TLI: Tucker-Lewis index
SRMR: Standardized root mean square residual
DF: Degrees of freedom
ICC: Intraclass correlation coefficient
SEM: Standard error of measurement
MDD: Minimum detectable difference
SD: Standard deviation
MI: Modification indices
BMI: Body Mass Index
