# Supplementary material for: Regular Physical Exercise Adherence Scale (REPEAS): a new instrument to measure environmental and personal barriers to adherence to regular physical exercise
Source: BMC Public Health. 2023 Dec 13;23:2491. doi: 10.1186/s12889-023-17438-1 (PMC10717144; doi:10.1186/s12889-023-17438-1)
Supplement: Supplementary file 1 — Additional file 1. [file 12889_2023_17438_MOESM1_ESM.pdf]

## Brazilian version of the Regular Physical Exercise Adherence Scale (REPEAS)

There are factors that hinder the practice of regular physical exercise. They can be personal, social or they can be related to the environmental and/or structural characteristics of the city where you live. Thus, mark below how much each item makes it difficult for you to adhere to the practice of physical exercise on a regular basis on a scale from 0 to 10, in which 0 represents "Does not hinder the practice of physical exercise" and 10 means "Makes it very difficult to practice physical exercise". Consider as regular practice that physical exercise performed 2 to 3 times a week for at least 3 months.

### ENVIRONMENTAL FACTORS DOMAIN

1. Climatic factors of the city where you live (for example, excessive heat or cold, rain, low humidity and/or others).

0 1 2 3 4 5 6 7 8 9 10

Does not hinder the practice of physical exercise — Makes it very difficult to exercise

2. Absence of suitable public places for the practice of physical exercise close to your residence (for example, squares, parks, fields, beaches and/or others).

0 1 2 3 4 5 6 7 8 9 10

Does not hinder the practice of physical exercise — Makes it very difficult to exercise

3. Feeling of insecurity in places close to home (for example, fear of being the victim of robberies, thefts and/or similar).

0 1 2 3 4 5 6 7 8 9 10

Does not hinder the practice of physical exercise — Makes it very difficult to exercise

4. Living far from appropriate places to practice physical exercise in your city.

0 1 2 3 4 5 6 7 8 9 10

Does not hinder the practice of physical exercise — Makes it very difficult to exercise

5. Difficulty accessing places to practice physical exercise due to the presence of uneven sidewalks, stairs and/or other obstacles.

0 1 2 3 4 5 6 7 8 9 10

Does not hinder the practice of physical exercise — Makes it very difficult to exercise

**PERSONAL FACTORS DOMAIN**

| 6. Lack of encouragement from family and/or friends. | | | | | | | | | | |
|---|---|---|---|---|---|---|---|---|---|---|
| 0 | 1 | 2 | 3 | 4 | 5 | 6 | 7 | 8 | 9 | 10 |
| Does not hinder the practice of physical exercise | | | | | | | | | | Makes it very difficult to exercise |

| 7. Limitations of one's own body (for example, presence of pain or difficulty moving around). | | | | | | | | | | |
|---|---|---|---|---|---|---|---|---|---|---|
| 0 | 1 | 2 | 3 | 4 | 5 | 6 | 7 | 8 | 9 | 10 |
| Does not hinder the practice of physical exercise | | | | | | | | | | Makes it very difficult to exercise |

| 8. Laziness, disinterest, discouragement and/or lack of disposition. | | | | | | | | | | |
|---|---|---|---|---|---|---|---|---|---|---|
| 0 | 1 | 2 | 3 | 4 | 5 | 6 | 7 | 8 | 9 | 10 |
| Does not hinder the practice of physical exercise | | | | | | | | | | Makes it very difficult to exercise |

| 9. Fear of getting injured or hurt. | | | | | | | | | | |
|---|---|---|---|---|---|---|---|---|---|---|
| 0 | 1 | 2 | 3 | 4 | 5 | 6 | 7 | 8 | 9 | 10 |
| Does not hinder the practice of physical exercise | | | | | | | | | | Makes it very difficult to exercise |

| 10. Lack of equipment, clothing, shoes and/or accessories for the practice of physical exercise. | | | | | | | | | | |
|---|---|---|---|---|---|---|---|---|---|---|
| 0 | 1 | 2 | 3 | 4 | 5 | 6 | 7 | 8 | 9 | 10 |
| Does not hinder the practice of physical exercise | | | | | | | | | | Makes it very difficult to exercise |

| 11. Lack of monitoring by a professional to advise you on the practice of physical exercise. | | | | | | | | | | |
|---|---|---|---|---|---|---|---|---|---|---|
| 0 | 1 | 2 | 3 | 4 | 5 | 6 | 7 | 8 | 9 | 10 |
| Does not hinder the practice of physical exercise | | | | | | | | | | Makes it very difficult to exercise |

| 12. Low self-esteem, shame, and/or other preoccupations with one's physical appearance. | | | | | | | | | | |
|---|---|---|---|---|---|---|---|---|---|---|
| 0 | 1 | 2 | 3 | 4 | 5 | 6 | 7 | 8 | 9 | 10 |
| Does not hinder the practice of physical exercise | | | | | | | | | | Makes it very difficult to exercise |

## Versão Brasileira do Regular Physical Exercise Adherence Scale (REPEAS)

Existem fatores que dificultam a prática de exercício físico regular. Eles podem ser pessoais, sociais ou podem estar relacionados às características ambientais e/ou estruturais da cidade onde você mora. Assim sendo, assinale abaixo quanto cada item dificulta a sua adesão à prática de exercício físico de forma regular em uma escala de 0 a 10, na qual 0 representa “Não dificulta a prática de exercício físico” e 10 significa “Dificulta muito a prática de exercício físico”. Considere como prática regular aquele exercício físico realizado de 2 a 3 vezes por semana durante no mínimo 3 meses.

**DOMÍNIO FATORES AMBIENTAIS**

1. Fatores climáticos da cidade onde reside (por exemplo, calor ou frio excessivo, chuva, baixa umidade e/ou outros).

0 1 2 3 4 5 6 7 8 9 10

Não dificulta a prática de exercício físico — Dificulta muito a prática de exercício físico

2. Ausência de locais públicos adequados para a prática de exercício físico próximo à sua residência (por exemplo, praças, parques, campos, praias e/ou outros).

0 1 2 3 4 5 6 7 8 9 10

Não dificulta a prática de exercício físico — Dificulta muito a prática de exercício físico

3. Sensação de insegurança nos locais perto da sua residência (por exemplo, medo de ser vítima de assaltos, furtos e/ou similares).

0 1 2 3 4 5 6 7 8 9 10

Não dificulta a prática de exercício físico — Dificulta muito a prática de exercício físico

4. Morar distante dos locais apropriados para a prática de exercício físico na sua cidade.

0 1 2 3 4 5 6 7 8 9 10

Não dificulta a prática de exercício físico — Dificulta muito a prática de exercício físico

5. Dificuldade de acesso aos locais para a prática de exercício físico devido à presença de calçadas irregulares, escadas e/ou outros obstáculos.

0 1 2 3 4 5 6 7 8 9 10

Não dificulta a prática de exercício físico — Dificulta muito a prática de exercício físico

**DOMÍNIO FATORES PESSOAIS**

| 6. Falta de incentivo da família e/ou amigos. | | | | | | | | | | |
|---|---|---|---|---|---|---|---|---|---|---|
| 0 | 1 | 2 | 3 | 4 | 5 | 6 | 7 | 8 | 9 | 10 |
| Não dificulta a prática de exercício físico | | | | | | | Dificulta muito a prática de exercício físico | | | |

| 7. Limitações do próprio corpo (por exemplo, presença de dor ou dificuldade para se locomover). | | | | | | | | | | |
|---|---|---|---|---|---|---|---|---|---|---|
| 0 | 1 | 2 | 3 | 4 | 5 | 6 | 7 | 8 | 9 | 10 |
| Não dificulta a prática de exercício físico | | | | | | | Dificulta muito a prática de exercício físico | | | |

| 8. Preguiça, desinteresse, desânimo e/ou falta de disposição. | | | | | | | | | | |
|---|---|---|---|---|---|---|---|---|---|---|
| 0 | 1 | 2 | 3 | 4 | 5 | 6 | 7 | 8 | 9 | 10 |
| Não dificulta a prática de exercício físico | | | | | | | Dificulta muito a prática de exercício físico | | | |

| 9. Medo de se lesionar ou se machucar. | | | | | | | | | | |
|---|---|---|---|---|---|---|---|---|---|---|
| 0 | 1 | 2 | 3 | 4 | 5 | 6 | 7 | 8 | 9 | 10 |
| Não dificulta a prática de exercício físico | | | | | | | Dificulta muito a prática de exercício físico | | | |

| 10. Falta de equipamentos, vestimentas, tênis e/ou acessórios para a prática de exercício físico. | | | | | | | | | | |
|---|---|---|---|---|---|---|---|---|---|---|
| 0 | 1 | 2 | 3 | 4 | 5 | 6 | 7 | 8 | 9 | 10 |
| Não dificulta a pratica de exercício físico | | | | | | | Dificulta muito a pratica de exercício físico | | | |

| 11. Falta de acompanhamento de um profissional para orientar sobre a prática de exercício físico. | | | | | | | | | | |
|---|---|---|---|---|---|---|---|---|---|---|
| 0 | 1 | 2 | 3 | 4 | 5 | 6 | 7 | 8 | 9 | 10 |
| Não dificulta a prática de exercício físico | | | | | | | Dificulta muito a prática de exercício físico | | | |

| 12. Baixa autoestima, vergonha e/ou outras preocupações com a própria aparência física. | | | | | | | | | | |
|---|---|---|---|---|---|---|---|---|---|---|
| 0 | 1 | 2 | 3 | 4 | 5 | 6 | 7 | 8 | 9 | 10 |
| Não dificulta a prática de exercício físico | | | | | | | Dificulta muito a prática de exercício físico | | | |
